# New patient-oriented summary measure of net total gain in certainty for dichotomous diagnostic tests

**DOI:** 10.1186/1742-5573-3-11

**Published:** 2006-10-05

**Authors:** Shai Linn, Peter D Grunau

**Affiliations:** 1 Faculty of Social Welfare and Health Studies, School of Public Health, University of Haifa, and Unit of Clinical Epidemiology, Rambam Medical Center, Haifa, Israel; 2 Faculty of Medicine, the University of British Columbia, Vancouver BC, Canada

## Abstract

**Objectives:**

To introduce a new, patient-oriented predictive index as a measure of gain in certainty.

**Study design:**

Algebraic equations.

**Results:**

A new measure is suggested based on error rates in a patient population. The new Predictive Summary Index (PSI) reflects the true total gain in certainty obtained by performing a diagnostic test based on knowledge of disease prevalence, i.e., the overall additional certainty. We show that the overall gain in certainty can be expressed in the form of the following expression: PSI = PPV+NPV-1. PSI is a more comprehensive measure than the post-test probability or the Youden Index (*J*). The reciprocal of *J *is interpreted as the number of persons with a given disease who need to be examined in order to detect correctly one person with the disease. The reciprocal of PSI is suggested as the number of persons who need to be examined in order to correctly predict a diagnosis of the disease.

**Conclusion:**

PSI provides more information than *J *and the predictive values, making it more appropriate in a clinical setting.

## Background

The main justification for performing a diagnostic test is to gain new information [1-3], beyond the existing probability (the prevalence) obtained from a positive test, i.e., prevalence minus the positive predictive value (PPV) and from a negative test, i.e., (1-prevalence) minus the negative predictive value (NPV). We introduce a predictive summary index (PSI), a new measure that summarizes the total gain in certainty, i.e., the overall additional certainty, expressed as PSI = PPV+NPV-1. We show that the reciprocal of PSI can be interpreted as the number of persons needed to be examined in order to correctly predict a diagnosis of the disease (NNP). We compare the PSI with a less informative summary measure of a test in a limited study population, the Youden Index (*J*), proposed by Youden [1] as a measure of the goodness of a diagnostic test.

### The terminology of diagnostic test characteristics [4-29]

Performance assessment of a dichotomous diagnostic test is usually based on assessing test performances in two different populations.

The first one is a selected study population of persons with and without a disease, in which both the diagnostic test and the definitive test (the gold standard) are evaluated, and for which sensitivity and specificity are calculated using a "sample truth table" [6] (Table 1). The second one is the general patient population to which the diagnostic test is applied. The experience of this population is summarized in a second 2 × 2 table (Table 2), which samples the data according to test status (positive or negative, i.e., pathological or normal result). It is the data in Table 2 that are of interest to the patient and the physician. However, it is often difficult to obtain the information needed to construct Table 2 because it is unfeasible or unethical to perform both the diagnostic tests and an additional definitive test in the general population to determine the true diagnosis according to the gold standard. Therefore, the positive predictive value (PPV) and the negative predictive value (NPV) are calculated from Table 1 using the Bayes' theorem.

**Table 1:** Two-by-two table for study results

		Gold Standard	
		
		S+	S-
Clinical Test	T+	a = True Positive	b = False Positive
	T-	c = False Negative	d = True Negative
	Total	a+c	b+d

1.1 *sensitivity *= *P*(*T*+|*S*+) = aa+c
 MathType@MTEF@5@5@+=feaafiart1ev1aqatCvAUfKttLearuWrP9MDH5MBPbIqV92AaeXatLxBI9gBaebbnrfifHhDYfgasaacH8akY=wiFfYdH8Gipec8Eeeu0xXdbba9frFj0=OqFfea0dXdd9vqai=hGuQ8kuc9pgc9s8qqaq=dirpe0xb9q8qiLsFr0=vr0=vr0dc8meaabaqaciaacaGaaeqabaqabeGadaaakeaadaWcaaqaaiabdggaHbqaaiabdggaHjabgUcaRiabdogaJbaaaaa@3184@

1.2 *specificity *= *P*(*T *- |*S*-) = db+d
 MathType@MTEF@5@5@+=feaafiart1ev1aqatCvAUfKttLearuWrP9MDH5MBPbIqV92AaeXatLxBI9gBaebbnrfifHhDYfgasaacH8akY=wiFfYdH8Gipec8Eeeu0xXdbba9frFj0=OqFfea0dXdd9vqai=hGuQ8kuc9pgc9s8qqaq=dirpe0xb9q8qiLsFr0=vr0=vr0dc8meaabaqaciaacaGaaeqabaqabeGadaaakeaadaWcaaqaaiabdsgaKbqaaiabdkgaIjabgUcaRiabdsgaKbaaaaa@318E@

1.3 *Positive Likelihood Ratio *= *PLR *= sensitivity1-specificity
 MathType@MTEF@5@5@+=feaafiart1ev1aqatCvAUfKttLearuWrP9MDH5MBPbIqV92AaeXatLxBI9gBaebbnrfifHhDYfgasaacH8akY=wiFfYdH8Gipec8Eeeu0xXdbba9frFj0=OqFfea0dXdd9vqai=hGuQ8kuc9pgc9s8qqaq=dirpe0xb9q8qiLsFr0=vr0=vr0dc8meaabaqaciaacaGaaeqabaqabeGadaaakeaadaWcaaqaaiabdohaZjabdwgaLjabd6gaUjabdohaZjabdMgaPjabdsha0jabdMgaPjabdAha2jabdMgaPjabdsha0jabdMha5bqaaiabigdaXiabc2caTiabdohaZjabdchaWjabdwgaLjabdogaJjabdMgaPjabdAgaMjabdMgaPjabdogaJjabdMgaPjabdsha0jabdMha5baaaaa@4D23@

1.4 *Negative Likelihood Ratio *= *NLR *= 1−sensitivityspecificity
 MathType@MTEF@5@5@+=feaafiart1ev1aqatCvAUfKttLearuWrP9MDH5MBPbIqV92AaeXatLxBI9gBaebbnrfifHhDYfgasaacH8akY=wiFfYdH8Gipec8Eeeu0xXdbba9frFj0=OqFfea0dXdd9vqai=hGuQ8kuc9pgc9s8qqaq=dirpe0xb9q8qiLsFr0=vr0=vr0dc8meaabaqaciaacaGaaeqabaqabeGadaaakeaadaWcaaqaaiabigdaXiabgkHiTiabdohaZjabdwgaLjabd6gaUjabdohaZjabdMgaPjabdsha0jabdMgaPjabdAha2jabdMgaPjabdsha0jabdMha5bqaaiabdohaZjabdchaWjabdwgaLjabdogaJjabdMgaPjabdAgaMjabdMgaPjabdogaJjabdMgaPjabdsha0jabdMha5baaaaa@4D2E@

Error terms for the study population:

α = false positive rate = *fpr *= bb+d
 MathType@MTEF@5@5@+=feaafiart1ev1aaatCvAUfKttLearuWrP9MDH5MBPbIqV92AaeXatLxBI9gBaebbnrfifHhDYfgasaacH8akY=wiFfYdH8Gipec8Eeeu0xXdbba9frFj0=OqFfea0dXdd9vqai=hGuQ8kuc9pgc9s8qqaq=dirpe0xb9q8qiLsFr0=vr0=vr0dc8meaabaqaciaacaGaaeqabaqabeGadaaakeaadaWcaaqaaiabbkgaIbqaaiabbkgaIHGaaiab=TcaRiabbsgaKbaaaaa@3186@

β = false negative rate = *fnr *= ca+c
 MathType@MTEF@5@5@+=feaafiart1ev1aaatCvAUfKttLearuWrP9MDH5MBPbIqV92AaeXatLxBI9gBaebbnrfifHhDYfgasaacH8akY=wiFfYdH8Gipec8Eeeu0xXdbba9frFj0=OqFfea0dXdd9vqai=hGuQ8kuc9pgc9s8qqaq=dirpe0xb9q8qiLsFr0=vr0=vr0dc8meaabaqaciaacaGaaeqabaqabeGadaaakeaadaWcaaqaaiabbogaJbqaaiabbggaHHGaaiab=TcaRiabbogaJbaaaaa@3184@

**Table 2:** Two-by-two table for a target population

		Gold Standard		
		
		S+	S-	Total
Clinical Test	T+	A = True Positive	B = False Positive	A+B
	T-	C = False Negative	D = True Negative	C+D

2.1 *PPV *= *P*(*S*+|*T*+) = AA+B
 MathType@MTEF@5@5@+=feaafiart1ev1aaatCvAUfKttLearuWrP9MDH5MBPbIqV92AaeXatLxBI9gBaebbnrfifHhDYfgasaacH8akY=wiFfYdH8Gipec8Eeeu0xXdbba9frFj0=OqFfea0dXdd9vqai=hGuQ8kuc9pgc9s8qqaq=dirpe0xb9q8qiLsFr0=vr0=vr0dc8meaabaqaciaacaGaaeqabaqabeGadaaakeaadaWcaaqaaiabdgeabbqaaiabdgeabjabgUcaRiabdkeacbaaaaa@30C1@

2.2 *NPV *= *P*(*S*-|*T*-) = DC+D
 MathType@MTEF@5@5@+=feaafiart1ev1aaatCvAUfKttLearuWrP9MDH5MBPbIqV92AaeXatLxBI9gBaebbnrfifHhDYfgasaacH8akY=wiFfYdH8Gipec8Eeeu0xXdbba9frFj0=OqFfea0dXdd9vqai=hGuQ8kuc9pgc9s8qqaq=dirpe0xb9q8qiLsFr0=vr0=vr0dc8meaabaqaciaacaGaaeqabaqabeGadaaakeaadaWcaaqaaiabdseaebqaaiabdoeadjabgUcaRiabdseaebaaaaa@30CF@

2.3 *PPR *= PPV1−NPV
 MathType@MTEF@5@5@+=feaafiart1ev1aaatCvAUfKttLearuWrP9MDH5MBPbIqV92AaeXatLxBI9gBaebbnrfifHhDYfgasaacH8akY=wiFfYdH8Gipec8Eeeu0xXdbba9frFj0=OqFfea0dXdd9vqai=hGuQ8kuc9pgc9s8qqaq=dirpe0xb9q8qiLsFr0=vr0=vr0dc8meaabaqaciaacaGaaeqabaqabeGadaaakeaadaWcaaqaaiabdcfaqjabdcfaqjabdAfawbqaaiabigdaXiabgkHiTiabd6eaojabdcfaqjabdAfawbaaaaa@35A3@[29]

2.4 *NPR *= 1−PPVNPV
 MathType@MTEF@5@5@+=feaafiart1ev1aaatCvAUfKttLearuWrP9MDH5MBPbIqV92AaeXatLxBI9gBaebbnrfifHhDYfgasaacH8akY=wiFfYdH8Gipec8Eeeu0xXdbba9frFj0=OqFfea0dXdd9vqai=hGuQ8kuc9pgc9s8qqaq=dirpe0xb9q8qiLsFr0=vr0=vr0dc8meaabaqaciaacaGaaeqabaqabeGadaaakeaadaWcaaqaaiabigdaXiabgkHiTiabdcfaqjabdcfaqjabdAfawbqaaiabd6eaojabdcfaqjabdAfawbaaaaa@35A3@[29]

Error terms for the target population:

α_*p *_= False Positive Rate = FPR = BA+B
 MathType@MTEF@5@5@+=feaafiart1ev1aaatCvAUfKttLearuWrP9MDH5MBPbIqV92AaeXatLxBI9gBaebbnrfifHhDYfgasaacH8akY=wiFfYdH8Gipec8Eeeu0xXdbba9frFj0=OqFfea0dXdd9vqai=hGuQ8kuc9pgc9s8qqaq=dirpe0xb9q8qiLsFr0=vr0=vr0dc8meaabaqaciaacaGaaeqabaqabeGadaaakeaadaWcaaqaaiabbkeacbqaaiabbgeabHGaaiab=TcaRiabbkeacbaaaaa@30C0@

β_*p *_= False Negative Rate = FNR = CC+D
 MathType@MTEF@5@5@+=feaafiart1ev1aaatCvAUfKttLearuWrP9MDH5MBPbIqV92AaeXatLxBI9gBaebbnrfifHhDYfgasaacH8akY=wiFfYdH8Gipec8Eeeu0xXdbba9frFj0=OqFfea0dXdd9vqai=hGuQ8kuc9pgc9s8qqaq=dirpe0xb9q8qiLsFr0=vr0=vr0dc8meaabaqaciaacaGaaeqabaqabeGadaaakeaadaWcaaqaaiabboeadbqaaiabboeadHGaaiab=TcaRiabbseaebaaaaa@30CA@

### 1. Definitions in the study population (Table 1) [4-29]

Sensitivity and specificity, and the likelihood ratio are frequently used measures (Table 1). False positive and false negative rates are often defined with reference to the study population. When diseased and non-diseased subjects are sampled, the false positive rate among persons without the disease is often defined as the α error (hence, *specificity *= 1 - α) and the false negative rate among persons with the disease is often defined as the β error (hence, *sensitivity *= 1 - β).

### 2. Definitions in the target (patient) population (Table 2) [4-29]

The measure of interest for physicians and patients alike is usually the positive predictive value (PPV) and the negative predictive value (NPV), (Table 2). When the prevalence P is known, the PPV can be derived from sensitivity and specificity.

In a previous publication [29] we proposed two new **ratio **measures in the patient population: the Positive Predictive Ration (PPR), which is analogous to the PLR, and the Negative Predictive Ration (NPR), which is analogous to the NLR. We do not discuss these ratio measures in the present article, and are mentioned only for better understanding the new **difference **measure proposed here.

Following Fleiss [27], Reid et al. [28], Knottnerus [22], and Riffenburgh [6], we use uppercase notations to define error rates in the patient (target) population (Table 2), in addition to the commonly used *fnr *and *fpr *defined above. Note that the interpretation of alpha and beta errors in the target population (Table 2) is different from that in the study population (Table 1); we therefore use the subscript *p *for errors in the target (patient) population.

### 3. The Youden index

In 1950, Youden [1] proposed the Youden Index as a measure of the goodness of a diagnostic test, using alpha and beta errors:

3.  *J *= 1 - (α + β)

If the test has no diagnostic value, the sensitivity = 1 - β equals the *fnr *= α, i.e., *J *= 0

(i.e., equal probability for disease among people with positive and negative test results).

If the test is always correct, all errors equal 0 and *J *= 1. Negative values of *J *(between -1 and 0) occur if the test is misleading, that is, if test results are negatively associated with the true diagnosis. Thus, as Pepe noted [26], *J *is a one-dimensional summary of test accuracy in the study population when the human and monetary costs associated with false positive errors in persons without the disease are similar in magnitude to those associated with false negative errors in persons with the disease [[26], p.80].

#### 3.1 Interpretation of the Youden index (*J*) in the study population

Another way of expressing this statistic in the study population is by means of sensitivity and specificity:

3.11 *J *= 1 - (α + β) = (1 - α) + (1 - β) - 1 = *sensitivity *+ *specificity *- 1

Assuming that sensitivity and specificity are equally important in determining the expected gain, the above equation implies that when sensitivity+specificity = 1, the test provides no overall information. Thus, if the test has no diagnostic value, e.g., the sensitivity and specificity are both 0.5, *J *= 0. If the test is always correct, the sensitivity and specificity total 2, and *J *= 1.

#### 3.2 Interpretation of the Youden index (*J*) as an average total net gain in certainty in the study population

As originally proposed by Youden [1], the index can be derived in another way: the net gain for persons with the disease can be defined as the difference between the percentage of persons diagnosed correctly (i.e., the sensitivity) and the *fnr*.

3.21 g+=aa+c−ca+c=tpr−fnr=a−ca+c
 MathType@MTEF@5@5@+=feaafiart1ev1aaatCvAUfKttLearuWrP9MDH5MBPbIqV92AaeXatLxBI9gBaebbnrfifHhDYfgasaacH8akY=wiFfYdH8Gipec8Eeeu0xXdbba9frFj0=OqFfea0dXdd9vqai=hGuQ8kuc9pgc9s8qqaq=dirpe0xb9q8qiLsFr0=vr0=vr0dc8meaabaqaciaacaGaaeqabaqabeGadaaakeaacqqGZaWmcqqGUaGlcqqGYaGmcqqGXaqmcqqGGaaicqWGNbWzdaWgaaWcbaGaey4kaScabeaakiabg2da9maalaaabaGaemyyaegabaGaemyyaeMaey4kaSIaem4yamgaaiabgkHiTmaalaaabaGaem4yamgabaGaemyyaeMaey4kaSIaem4yamgaaiabg2da9iabdsha0jabdchaWjabdkhaYjabgkHiTiabdAgaMjabd6gaUjabdkhaYjabg2da9maalaaabaGaemyyaeMaeyOeI0Iaem4yamgabaGaemyyaeMaey4kaSIaem4yamgaaaaa@51A5@

Similarly, the net gain for persons without the disease can be defined as the difference between the percentage of persons diagnosed correctly without a disease (i.e., the specificity) and the *fpr*.

3.22 g−=tnr−fpr=dd+b−bd+b=d−bd+b
 MathType@MTEF@5@5@+=feaafiart1ev1aaatCvAUfKttLearuWrP9MDH5MBPbIqV92AaeXatLxBI9gBaebbnrfifHhDYfgasaacH8akY=wiFfYdH8Gipec8Eeeu0xXdbba9frFj0=OqFfea0dXdd9vqai=hGuQ8kuc9pgc9s8qqaq=dirpe0xb9q8qiLsFr0=vr0=vr0dc8meaabaqaciaacaGaaeqabaqabeGadaaakeaacqqGZaWmcqqGUaGlcqqGYaGmcqqGYaGmcqqGGaaicqWGNbWzdaWgaaWcbaGaeyOeI0cabeaakiabg2da9iabdsha0jabd6gaUjabdkhaYjabgkHiTiabdAgaMjabdchaWjabdkhaYjabg2da9maalaaabaGaemizaqgabaGaemizaqMaey4kaSIaemOyaigaaiabgkHiTmaalaaabaGaemOyaigabaGaemizaqMaey4kaSIaemOyaigaaiabg2da9maalaaabaGaemizaqMaeyOeI0IaemOyaigabaGaemizaqMaey4kaSIaemOyaigaaaaa@51C6@

Assuming that the value gain in certainty for these two populations (persons with and without the disease) is similar, and that false positives are as undesirable as false negatives, the unweighted average of the two measures is *J *(see appendix A):

3.23g++g−2=12[a−ca+c+d−bd+b]=J
 MathType@MTEF@5@5@+=feaafiart1ev1aqatCvAUfKttLearuWrP9MDH5MBPbIqV92AaeXatLxBI9gBaebbnrfifHhDYfgasaacH8akY=wiFfYdH8Gipec8Eeeu0xXdbba9frFj0=OqFfea0dXdd9vqai=hGuQ8kuc9pgc9s8qqaq=dirpe0xb9q8qiLsFr0=vr0=vr0dc8meaabaqaciaacaGaaeqabaqabeGadaaakeaacqaIZaWmcqGGUaGlcqaIYaGmcqaIZaWmdaWcaaqaaiabdEgaNnaaBaaaleaacqGHRaWkaeqaaOGaey4kaSIaem4zaC2aaSbaaSqaaiabgkHiTaqabaaakeaacqaIYaGmaaGaeyypa0ZaaSaaaeaacqaIXaqmaeaacqaIYaGmaaGaei4waS1aaSaaaeaacqWGHbqycqGHsislcqWGJbWyaeaacqWGHbqycqGHRaWkcqWGJbWyaaGaey4kaSYaaSaaaeaacqWGKbazcqGHsislcqWGIbGyaeaacqWGKbazcqGHRaWkcqWGIbGyaaGaeiyxa0Laeyypa0JaemOsaOeaaa@4DE3@

#### 3.3 Analogies to a cohort study

*J *can also be interpreted as the difference between the true and false positive rates.

3.31 J=(1−β)−α=sensitivity−fpr=aa+c−bb+d
 MathType@MTEF@5@5@+=feaafiart1ev1aqatCvAUfKttLearuWrP9MDH5MBPbIqV92AaeXatLxBI9gBaebbnrfifHhDYfgasaacH8akY=wiFfYdH8Gipec8Eeeu0xXdbba9frFj0=OqFfea0dXdd9vqai=hGuQ8kuc9pgc9s8qqaq=dirpe0xb9q8qiLsFr0=vr0=vr0dc8meaabaqaciaacaGaaeqabaqabeGadaaakeaacqqGZaWmcqqGUaGlcqqGZaWmcqqGXaqmcqqGGaaicqWGkbGscqGH9aqpcqGGOaakcqGHXaqmcqGHsislcqaHYoGycqGGPaqkcqGHsislcqaHXoqycqGH9aqpcqWGZbWCcqWGLbqzcqWGUbGBcqWGZbWCcqWGPbqAcqWG0baDcqWGPbqAcqWG2bGDcqWGPbqAcqWG0baDcqWG5bqEcqGHsislcqWGMbGzcqWGWbaCcqWGYbGCcqGH9aqpdaWcaaqaaiabdggaHbqaaiabdggaHjabgUcaRiabdogaJbaacqGHsisldaWcaaqaaiabdkgaIbqaaiabdkgaIjabgUcaRiabdsgaKbaaaaa@5C35@

Thus, *J *reflects the *excess *of the proportion of a *positive *result among patients with vs. patients without the disease [24-26].

This interpretation of *J *is analogous to a commonly used measure in cohort studies, the rate (risk) difference (RD). Counter-intuitively, the analogy is to a cohort study rather than to a case control study, although in a study population the compared study groups are persons with and without the disease because the "causative" variable (i.e., the "exposure") is the fact that a person does or does not have the disease. The diagnostic test results (positive or negative) are the "outcome" of the disease.

*J *can also be written as:

3.32 J=(1−α)−β=specificity−fnr=db+d−ca+c
 MathType@MTEF@5@5@+=feaafiart1ev1aqatCvAUfKttLearuWrP9MDH5MBPbIqV92AaeXatLxBI9gBaebbnrfifHhDYfgasaacH8akY=wiFfYdH8Gipec8Eeeu0xXdbba9frFj0=OqFfea0dXdd9vqai=hGuQ8kuc9pgc9s8qqaq=dirpe0xb9q8qiLsFr0=vr0=vr0dc8meaabaqaciaacaGaaeqabaqabeGadaaakeaacqqGZaWmcqqGUaGlcqqGZaWmcqqGYaGmcqqGGaaicqWGkbGscqGH9aqpcqGGOaakcqGHXaqmcqGHsislcqaHXoqycqGGPaqkcqGHsislcqaHYoGycqGH9aqpcqWGZbWCcqWGWbaCcqWGLbqzcqWGJbWycqWGPbqAcqWGMbGzcqWGPbqAcqWGJbWycqWGPbqAcqWG0baDcqWG5bqEcqGHsislcqWGMbGzcqWGUbGBcqWGYbGCcqGH9aqpdaWcaaqaaiabdsgaKbqaaiabdkgaIjabgUcaRiabdsgaKbaacqGHsisldaWcaaqaaiabdogaJbqaaiabdggaHjabgUcaRiabdogaJbaaaaa@5BDD@

Thus, *J *also reflects the excess in the proportion of a *negative *result among patients *without *vs. patients with the disease [25].

This interpretation of *J *is analogous to a rate difference of having *no *disease, when the focus of the investigation is the absence of a disease. Alternatively, it can be analogous to a follow-up study that defines better health as the outcome. In other words, it is analogous to the rate difference of a protective agent, such as vaccination, when better health and disease are compared as outcomes.

A further analogy of 1J
 MathType@MTEF@5@5@+=feaafiart1ev1aqatCvAUfKttLearuWrP9MDH5MBPbIqV92AaeXatLxBI9gBaebbnrfifHhDYfgasaacH8akY=wiFfYdH8Gipec8Eeeu0xXdbba9frFj0=OqFfea0dXdd9vqai=hGuQ8kuc9pgc9s8qqaq=dirpe0xb9q8qiLsFr0=vr0=vr0dc8meaabaqaciaacaGaaeqabaqabeGadaaakeaadaWcaaqaaiabigdaXaqaaiabdQeakbaaaaa@2ECA@ to the well-known measure of the "number needed to treat" (NNT) = 1RD
 MathType@MTEF@5@5@+=feaafiart1ev1aqatCvAUfKttLearuWrP9MDH5MBPbIqV92AaeXatLxBI9gBaebbnrfifHhDYfgasaacH8akY=wiFfYdH8Gipec8Eeeu0xXdbba9frFj0=OqFfea0dXdd9vqai=hGuQ8kuc9pgc9s8qqaq=dirpe0xb9q8qiLsFr0=vr0=vr0dc8meaabaqaciaacaGaaeqabaqabeGadaaakeaadaWcaaqaaiabigdaXaqaaiabdkfasjabdseaebaaaaa@2FEB@ is in order. Thus, 1J
 MathType@MTEF@5@5@+=feaafiart1ev1aqatCvAUfKttLearuWrP9MDH5MBPbIqV92AaeXatLxBI9gBaebbnrfifHhDYfgasaacH8akY=wiFfYdH8Gipec8Eeeu0xXdbba9frFj0=OqFfea0dXdd9vqai=hGuQ8kuc9pgc9s8qqaq=dirpe0xb9q8qiLsFr0=vr0=vr0dc8meaabaqaciaacaGaaeqabaqabeGadaaakeaadaWcaaqaaiabigdaXaqaaiabdQeakbaaaaa@2ECA@ may be interpreted as the number of patients needed to be examined in order to correctly detect (NND, Table 3) one person with the disease in a study population (Table 1) of persons with and without the known disease.

**Table 3:** Analyses of data in an example by Sackett et al [8 (Table 4–10, pp. 95–98)].

	*Patient A*: High prior suspicion of coronary disease	*Patient B*: Low prior suspicion of coronary disease	*Patient C*: Intermediate prior suspicion of coronary disease
Prevalence	90%	5%	50%
A	540	30	300
B	9	86	45
C	360	20	200
D	91	864	455
PPV	540/549 = 98.36%	30/116 = 25.86%	300/345 = 87%
NPV	91/451 = 20.18%	864/884 = 97.74%	455/655 = 69.47%
FPR	9/549 = 1.64%	86/116 = 74.14%	45/345 = 13.04%
FNR	360/451 = 79.82%	20/884 = 2.26%	200/655 = 30.53
PLR	6.67	6.67	6.67
*J *= sensitivity+specificity-1	51.41%	51.41%	51.41%
NND = 1/*J*	1.95	1.95	1.95
PSI = Ψ = PPV+NPV-1 = = [PPV-Prevalence]+ [NPV-(1-Prevalence)]	18.54%	23.60%	56.47%
NNP = 1/PSI = 1/Ψ	5.4	4.2	1.8

The use of exercise ECG with three types of patients with prior coronary disease probability of 5%, 90% and 50%, using angiogram as a gold standard.

The commonly used PLR is a ratio measure, analogous to the risk ratio (RR) in a follow-up study. Thus, *J *and PLR describe a diagnostic test in the study population of Table 1 only, in two different dimensions.

### 4. The new measure in the target population: Predictive Summary Index (PSI, Ψ)

Because the Youden Index (*J*) is based on the study population (Table 1) [25,26], it does not convey information about the specific clinical setting in which the diagnostic test is being applied. Patients and physicians are more interested in a similar Predictive Summary Index (PSI, Ψ) in the target population (Table 2). Because the interpretation of alpha and beta errors in the two populations is different, *J *and PSI have different underlying interpretations.

PSI can be derived in the target (patient) population as a measure of the goodness of the predictability in a diagnostic test, using alpha and beta errors in the target population:

4. PSI = *PPV *+ *NPV *- 1 = 1 - (α_*p *_+ β_*p*_)

If the test has no predictive value, PPV equals *FNR*, i.e., there is an equal probability for disease among people with positive and negative test results (Table 2). Hence:

1 - α_*p *_= β_*p*_

*thus*,

PSI = 0

If the test is always correct, all errors equal 0 and PSI = 1. Negative values of PSI (between -1 and 0) occur if the test is misleading, i.e., occurrence of disease is negatively associated with tests results.

#### 4.1 Interpretation of PSI in the study population as a true (total) gain in certainty

Another way of expressing this statistic in the study population is by PPV and NPV:

4.11 predictive index = PI = 1 - (α_*p *_+ β_*p*_) = (1 - α_*p*_) + (1 - β_*p*_) - 1 = *PPV *+ *NPV *- 1

*i.e*.,

4.111 PSI=AA+B+DC+D−1
 MathType@MTEF@5@5@+=feaafiart1ev1aaatCvAUfKttLearuWrP9MDH5MBPbIqV92AaeXatLxBI9gBaebbnrfifHhDYfgasaacH8akY=wiFfYdH8Gipec8Eeeu0xXdbba9frFj0=OqFfea0dXdd9vqai=hGuQ8kuc9pgc9s8qqaq=dirpe0xb9q8qiLsFr0=vr0=vr0dc8meaabaqaciaacaGaaeqabaqabeGadaaakeaacqqG0aancqqGUaGlcqqGXaqmcqqGXaqmcqqGXaqmcqqGGaaicqqGqbaucqqGtbWucqqGjbqscqGH9aqpdaWcaaqaaiabdgeabbqaaiabdgeabjabgUcaRiabdkeacbaacqGHRaWkdaWcaaqaaiabdseaebqaaiabdoeadjabgUcaRiabdseaebaacqGHsislcqaIXaqmaaa@416A@

This can be expressed using the data in the study population (Table 1) by means of the Bayes' theorem.

4.12 PSI=sensitivity*P sensitivity*P + (1−specificity)*(1−P)+specificity*(1−P) specificity*(1−P) + (1−sensitivity)*P−1
 MathType@MTEF@5@5@+=feaafiart1ev1aaatCvAUfKttLearuWrP9MDH5MBPbIqV92AaeXatLxBI9gBaebbnrfifHhDYfgasaacH8akY=wiFfYdH8Gipec8Eeeu0xXdbba9frFj0=OqFfea0dXdd9vqai=hGuQ8kuc9pgc9s8qqaq=dirpe0xb9q8qiLsFr0=vr0=vr0dc8meaabaqaciaacaGaaeqabaqabeGadaaakeaacqqG0aancqqGUaGlcqqGXaqmcqqGYaGmcqqGGaaicqqGqbaucqqGtbWucqqGjbqscqGH9aqpdaWcaaqaaiabdohaZjabdwgaLjabd6gaUjabdohaZjabdMgaPjabdsha0jabdMgaPjabdAha2jabdMgaPjabdsha0jabdMha5jabcQcaQiabdcfaqbqaaiabbccaGiabdohaZjabdwgaLjabd6gaUjabdohaZjabdMgaPjabdsha0jabdMgaPjabdAha2jabdMgaPjabdsha0jabdMha5jabcQcaQiabdcfaqjabbccaGiabgUcaRiabbccaGiabcIcaOiabigdaXiabgkHiTiabdohaZjabdchaWjabdwgaLjabdogaJjabdMgaPjabdAgaMjabdMgaPjabdogaJjabdMgaPjabdsha0jabdMha5jabcMcaPiabcQcaQiabcIcaOiabigdaXiabgkHiTiabdcfaqjabcMcaPaaacqGHRaWkdaWcaaqaaiabdohaZjabdchaWjabdwgaLjabdogaJjabdMgaPjabdAgaMjabdMgaPjabdogaJjabdMgaPjabdsha0jabdMha5jabcQcaQiabcIcaOiabigdaXiabgkHiTiabdcfaqjabcMcaPaqaaiabbccaGiabdohaZjabdchaWjabdwgaLjabdogaJjabdMgaPjabdAgaMjabdMgaPjabdogaJjabdMgaPjabdsha0jabdMha5jabcQcaQiabcIcaOiabigdaXiabgkHiTiabdcfaqjabcMcaPiabbccaGiabgUcaRiabbccaGiabcIcaOiabigdaXiabgkHiTiabdohaZjabdwgaLjabd6gaUjabdohaZjabdMgaPjabdsha0jabdMgaPjabdAha2jabdMgaPjabdsha0jabdMha5jabcMcaPiabcQcaQiabdcfaqbaacqGHsislcqaIXaqmaaa@B87E@

PSI in the target population is a generalization of the measure of gain in certainty from a diagnostic test, as proposed by Connell and Koepsell [2].

Physicians can guess the probability of a disease without performing a diagnostic test based on prevalence (the prior probability of a disease). A true gain in the certainty that a disease is present occurs when the posterior probability (the PPV) is greater than the prior probability (the prevalence). A true gain in the certainty that there is no disease occurs when the posterior probability of no disease (the NPV) is greater than the prior probability of no disease (1-prevalence).

The *total net gain *in certainty is a summation of these gains. Algebraically, it is the PSI.

4.13 Total net gain in certainty =

= [*PPV *- Prevalence] + [NPV - (1 - Prevalence)] =

= PPV+NPV-1 = predictive index = PSI

Equation 4.13 is valid when the human and monetary costs associated with the false positive errors of a diagnostic test are similar in magnitude to those associated with false negative errors. PSI in the patient population can thus be interpreted analogously to the Zhou et al. [25] and Pepe [26] interpretations of *J *in the study population, as a one-dimensional summary of "test predictability" [[26], p.80].

#### 4.2 Interpretation of PSI in the target population as the average net gain in certainty for persons with a positive or negative test result

PSI can be derived in another way as follows: the net gain in certainty for persons in the target population with a positive test result is the difference between the percentage of persons predicted correctly to have the disease the PPV) and the *FPR*.

4.21 G+=AA+B−BA+B=PPV−FPR=A−BA+B
 MathType@MTEF@5@5@+=feaafiart1ev1aaatCvAUfKttLearuWrP9MDH5MBPbIqV92AaeXatLxBI9gBaebbnrfifHhDYfgasaacH8akY=wiFfYdH8Gipec8Eeeu0xXdbba9frFj0=OqFfea0dXdd9vqai=hGuQ8kuc9pgc9s8qqaq=dirpe0xb9q8qiLsFr0=vr0=vr0dc8meaabaqaciaacaGaaeqabaqabeGadaaakeaacqaI0aancqGGUaGlcqaIYaGmcqaIXaqmcqqGGaaicqWGhbWrdaWgaaWcbaGaey4kaScabeaakiabg2da9maalaaabaGaemyqaeeabaGaemyqaeKaey4kaSIaemOqaieaaiabgkHiTmaalaaabaGaemOqaieabaGaemyqaeKaey4kaSIaemOqaieaaiabg2da9iabdcfaqjabdcfaqjabdAfawjabgkHiTiabdAeagjabdcfaqjabdkfasjabg2da9maalaaabaGaemyqaeKaeyOeI0IaemOqaieabaGaemyqaeKaey4kaSIaemOqaieaaaaa@4D77@

Similarly, the net gain for persons with a negative test result is the difference between the percentage of persons predicted correctly to be without the disease (the NPV) and the *FNR*.

4.22 G−=DC+D−CC+D=NPV−FNR=D−CC+D
 MathType@MTEF@5@5@+=feaafiart1ev1aaatCvAUfKttLearuWrP9MDH5MBPbIqV92AaeXatLxBI9gBaebbnrfifHhDYfgasaacH8akY=wiFfYdH8Gipec8Eeeu0xXdbba9frFj0=OqFfea0dXdd9vqai=hGuQ8kuc9pgc9s8qqaq=dirpe0xb9q8qiLsFr0=vr0=vr0dc8meaabaqaciaacaGaaeqabaqabeGadaaakeaacqaI0aancqGGUaGlcqaIYaGmcqaIYaGmcqqGGaaicqWGhbWrdaWgaaWcbaGaeyOeI0cabeaakiabg2da9maalaaabaGaemiraqeabaGaem4qamKaey4kaSIaemiraqeaaiabgkHiTmaalaaabaGaem4qameabaGaem4qamKaey4kaSIaemiraqeaaiabg2da9iabd6eaojabdcfaqjabdAfawjabgkHiTiabdAeagjabd6eaojabdkfasjabg2da9maalaaabaGaemiraqKaeyOeI0Iaem4qameabaGaem4qamKaey4kaSIaemiraqeaaaaa@4DA4@

Assuming that the value gain in certainty for these two populations (persons with positive and negative test results) is similar, and that false positives (FPR) are as undesirable as false negatives (FNR), the unweighted average of the two gains is PSI (Appendix B):

4.23 G++G−2=12[A−BA+B+D−CD+C]
 MathType@MTEF@5@5@+=feaafiart1ev1aqatCvAUfKttLearuWrP9MDH5MBPbIqV92AaeXatLxBI9gBaebbnrfifHhDYfgasaacH8akY=wiFfYdH8Gipec8Eeeu0xXdbba9frFj0=OqFfea0dXdd9vqai=hGuQ8kuc9pgc9s8qqaq=dirpe0xb9q8qiLsFr0=vr0=vr0dc8meaabaqaciaacaGaaeqabaqabeGadaaakeaacqaI0aancqGGUaGlcqaIYaGmcqaIZaWmcqqGGaaidaWcaaqaaiabbEeahnaaBaaaleaacqqGRaWkaeqaaOGaey4kaSIaem4raC0aaSbaaSqaaiabgkHiTaqabaaakeaacqaIYaGmaaGaeyypa0ZaaSaaaeaacqaIXaqmaeaacqaIYaGmaaGaei4waS1aaSaaaeaacqWGbbqqcqGHsislcqWGcbGqaeaacqWGbbqqcqGHRaWkcqWGcbGqaaGaey4kaSYaaSaaaeaacqWGebarcqGHsislcqWGdbWqaeaacqWGebarcqGHRaWkcqWGdbWqaaGaeiyxa0faaa@4A02@

#### 4.3 Analogies to a case control study

The PSI can be interpreted as the difference between the correct prediction of a disease by the test and a false negative result of the test in the target population.

4.31 PSI=(1−αp)−βp=PPV−FNR=AA+B−CC+D
 MathType@MTEF@5@5@+=feaafiart1ev1aqatCvAUfKttLearuWrP9MDH5MBPbIqV92AaeXatLxBI9gBaebbnrfifHhDYfgasaacH8akY=wiFfYdH8Gipec8Eeeu0xXdbba9frFj0=OqFfea0dXdd9vqai=hGuQ8kuc9pgc9s8qqaq=dirpe0xb9q8qiLsFr0=vr0=vr0dc8meaabaqaciaacaGaaeqabaqabeGadaaakeaacqqG0aancqqGUaGlcqqGZaWmcqqGXaqmcqqGGaaicqqGqbaucqqGtbWucqqGjbqscqGH9aqpcqGGOaakcqGHXaqmcqGHsislcqaHXoqydaWgaaWcbaGaemiCaahabeaakiabcMcaPiabgkHiTiabek7aInaaBaaaleaacqWGWbaCaeqaaOGaeyypa0JaemiuaaLaemiuaaLaemOvayLaeyOeI0IaemOrayKaemOta4KaemOuaiLaeyypa0ZaaSaaaeaacqWGbbqqaeaacqWGbbqqcqGHRaWkcqWGcbGqaaGaeyOeI0YaaSaaaeaacqWGdbWqaeaacqWGdbWqcqGHRaWkcqWGebaraaaaaa@5391@

Thus, PSI reflects the excess in the proportion of the disease when the test yields a positive result vs. the proportion of the disease when the test is negative, similar to the Zhou et al. [25] interpretation of *J *in the study population.

This interpretation of PSI is analogous to the exposure rate difference, an uncommon measure of no interest in case control studies. Counter-intuitively, the analogy is to a case control study rather than to a follow-up study, although the compared study groups are persons with positive vs. negative test results. As mentioned above, the "causative" variable (the "exposure") is the fact that a person does or does not have the disease. The diagnostic test results (positive or negative) are the "outcome" of the disease.

Although in case control studies there is no interest in the exposure rate difference, where we are interested in the association of exposure with a resulting disease, the PSI, analogous to the exposure rate difference, *is *of interest in clinical epidemiology in the context of the data in Table 2, i.e., in the target population.

We suggest using a new statistic, NNP = 1PSI
 MathType@MTEF@5@5@+=feaafiart1ev1aqatCvAUfKttLearuWrP9MDH5MBPbIqV92AaeXatLxBI9gBaebbnrfifHhDYfgasaacH8akY=wiFfYdH8Gipec8Eeeu0xXdbba9frFj0=OqFfea0dXdd9vqai=hGuQ8kuc9pgc9s8qqaq=dirpe0xb9q8qiLsFr0=vr0=vr0dc8meaabaqaciaacaGaaeqabaqabeGadaaakeaadaWcaaqaaiabigdaXaqaaiabbcfaqjabbofatjabbMeajbaaaaa@311A@, analogously to NND to estimate the number of patients needed to be examined in the patient population (Table 2) in order to correctly identify (predict) the positive diagnosis of one person. For example, this can be the number of people who would have to undergo exercise ECG to correctly identify one person who would eventually be diagnosed by angiography as having coronary artery disease. This measure may be abbreviated as the "number needed to predict," or NNP.

Similarly, one can also interpret PSI as:

4.32 PSI=(1−βp)−αp=NPV−FPR=DC+D−BA+B
 MathType@MTEF@5@5@+=feaafiart1ev1aqatCvAUfKttLearuWrP9MDH5MBPbIqV92AaeXatLxBI9gBaebbnrfifHhDYfgasaacH8akY=wiFfYdH8Gipec8Eeeu0xXdbba9frFj0=OqFfea0dXdd9vqai=hGuQ8kuc9pgc9s8qqaq=dirpe0xb9q8qiLsFr0=vr0=vr0dc8meaabaqaciaacaGaaeqabaqabeGadaaakeaacqaI0aancqGGUaGlcqaIZaWmcqaIYaGmcqqGGaaicqqGqbaucqqGtbWucqqGjbqscqGH9aqpcqGGOaakcqGHXaqmcqGHsislcqaHYoGydaWgaaWcbaGaemiCaahabeaakiabcMcaPiabgkHiTiabeg7aHnaaBaaaleaacqWGWbaCaeqaaOGaeyypa0JaemOta4KaemiuaaLaemOvayLaeyOeI0IaemOrayKaemiuaaLaemOuaiLaeyypa0ZaaSaaaeaacqWGebaraeaacqWGdbWqcqGHRaWkcqWGebaraaGaeyOeI0YaaSaaaeaacqWGcbGqaeaacqWGbbqqcqGHRaWkcqWGcbGqaaaaaa@53AD@

Thus, PSI reflects the excess in the proportion of no disease when the test yields a negative result vs. no disease when the test is positive, similar to the Zhou et al. [24] interpretation of *J*.

This interpretation of PSI is analogous to the exposure rate difference when the lack of exposure is the focus of the investigation, and it is compared with exposure to the causative agent.

NNP measures also the number of patients needed to be examined in the patient population in order to correctly identify (predict) the negative diagnosis of one person.

### 5. Example using published data

Consider the example provided by Sackett et al. [[8], p. 95–98] on the importance of prevalence for the evaluation by exercise ECG of three types of patients with prior coronary disease probability of 5%, 90%, and 50%, using angiogram as a gold standard (Table 3). Originally, the example was designed to demonstrate the importance of prevalence in determining the PPV and NPV of a diagnostic test (exercise ECG). The sensitivity of the ECG was 60.35% and the specificity 91.06%. Thus the Youden Index was *J *= 51.41% for all three types of patients.

As indicated by Sackett et al. [8], patient C, with a 50% prior probability of the disease, can benefit more from the test than patients A and B. But both the PLR and *J *statistics (in the study population) are identical for the three types of patients and do not convey this information. However, PSI statistics provide the information relevant to patients and physicians, in this case a PSI of 18.54%, 23.6%, and 56.47% for patient populations with a prevalence of 90%, 5%, and 50%. PSI is a comprehensive measure that conveys information about prior probabilities of a disease (the prevalence) together with the information about the posterior probability, after performing the diagnostic test, the PPV, as well as the probability of no disease (1-prevalence), and the NPV.

While NND = 1J
 MathType@MTEF@5@5@+=feaafiart1ev1aqatCvAUfKttLearuWrP9MDH5MBPbIqV92AaeXatLxBI9gBaebbnrfifHhDYfgasaacH8akY=wiFfYdH8Gipec8Eeeu0xXdbba9frFj0=OqFfea0dXdd9vqai=hGuQ8kuc9pgc9s8qqaq=dirpe0xb9q8qiLsFr0=vr0=vr0dc8meaabaqaciaacaGaaeqabaqabeGadaaakeaadaWcaaqaaiabigdaXaqaaiabdQeakbaaaaa@2ECA@ remains constant irrespective of prevalence, NNP = 1PSI
 MathType@MTEF@5@5@+=feaafiart1ev1aqatCvAUfKttLearuWrP9MDH5MBPbIqV92AaeXatLxBI9gBaebbnrfifHhDYfgasaacH8akY=wiFfYdH8Gipec8Eeeu0xXdbba9frFj0=OqFfea0dXdd9vqai=hGuQ8kuc9pgc9s8qqaq=dirpe0xb9q8qiLsFr0=vr0=vr0dc8meaabaqaciaacaGaaeqabaqabeGadaaakeaadaWcaaqaaiabigdaXaqaaiabbcfaqjabbofatjabbMeajbaaaaa@311A@ is dependent on prevalence and yields values of 5.4, 4.2, and 1.8 for patient populations with a prevalence of 90%, 5%, and 50%. The range of NNP demonstrates that the exercise test is most efficient when the prevalence is 50%, as Sackett et al. [8] claimed. Only two patients would be needed to show a valuable information gain from an exercise test for predicting coronary heart disease when the prevalence is 50%, compared with more than 5 patients when the prevalence is 90%.

## Discussion

Most of the classic epidemiology textbooks do not discuss the Youden Index, probably because its utility is limited, as shown in the above example: it remains unchanged in populations with different prevalence of the disease. Similarly, only few textbooks discuss measures of gain in certainty despite the fact that the purpose of a diagnostic test is to reach a better diagnosis by a gain in certainty. We suggest a simple and informative summary measure of the total gain in certainty, the PSI, which is readily calculable from 2 × 2 tables that describe the performance of a diagnostic test in the patient (target) population. We suggest to use the capital Greek letter *PSI *(Ψ) for this index.

PSI is of interest to patients: it describes how much more likely the patient is to be correctly diagnosed with a disease after a positive test, and how much more likely the patient is not to be incorrectly diagnosed with a disease after a negative test. This information may be critical. PSI can serve as an indicator for the possible use of the results of a specific test, and makes possible comparisons between tests within the context of prior probabilities: the higher the PSI, the more informative the test is for patients and physicians.

*J *is a descriptor of a diagnostic test among theoretical groups of persons with and without a disease. PSI is a descriptor of test performance for persons who test positive or negative. Thus, PSI reflects the total net gain in certainty resulting from a diagnostic test in clinical conditions, which is of interest to physicians and patients alike. This information is not available through *J *or any of the diagnostic test characteristics, including sensitivity, specificity (and thereby the likelihood ratios), and PPV.

PSI is similar in form to *J *but not identical with it. *J *is depends entirely on sensitivity and specificity. By contrast, PSI is partially dependent on those parameters and on the prevalence. Although Connell and Koepsell [2] advocated the use of a measure similar to PSI in the patient population, they explored only a special case in which the study population and the target population are identical and the prevalence is known from the study population.

prevalence=a+ca+b+c+d
 MathType@MTEF@5@5@+=feaafiart1ev1aqatCvAUfKttLearuWrP9MDH5MBPbIqV92AaeXatLxBI9gBaebbnrfifHhDYfgasaacH8akY=wiFfYdH8Gipec8Eeeu0xXdbba9frFj0=OqFfea0dXdd9vqai=hGuQ8kuc9pgc9s8qqaq=dirpe0xb9q8qiLsFr0=vr0=vr0dc8meaabaqaciaacaGaaeqabaqabeGadaaakeaacqWGWbaCcqWGYbGCcqWGLbqzcqWG2bGDcqWGHbqycqWGSbaBcqWGLbqzcqWGUbGBcqWGJbWycqWGLbqzcqGH9aqpdaWcaaqaaiabdggaHjabgUcaRiabdogaJbqaaiabdggaHjabgUcaRiabdkgaIjabgUcaRiabdogaJjabgUcaRiabdsgaKbaaaaa@46C1@

This is a specific condition that seldom occurs. PSI is not limited to this specific situation.

Increasing diagnostic power through new technologies usually serves to increase the sensitivity of diagnostic procedures, with a potential for higher *J*. But new technologies can also decrease specificity and create false positive findings, resulting in anxiety and unnecessary costs [30-34]. These are not measurable by *J *in a limited study population. Therefore, PSI should be evaluated for each new diagnostic test to estimate its diagnostic accuracy in the patient population.

When physicians have data on the patient population and can construct the appropriate Table 2 for the clinical (target) population, a clinic-specific PSI can be calculated directly from the patient population. In a clinical setting, the calculation of PSI does not depend on knowing the prevalence, a statistic that is often not available for specific patient populations. Following up on patients with positive or negative tests will yield directly the PPV and NPV without the need for sensitivity, specificity, or Bayes' theorem. PSI calculations are readily available from the PPV and NPV. For example, sonographers can follow up on fetuses who test positive or negative for malformations and determine the PPV and NPV in their prenatal clinic [29]. Their PSI would serve to indicate the net gain in certainty for their patients (equation 4.13) and the average net gain in information for patients who test positive or negative (4.23). When sensitivity, specificity, and external knowledge of the prevalence are available, Bayes' theorem can be used to calculate the PPV, the NPV, and thereby the PSI.

Moons and Harrell have recently criticized the use of sensitivity or specificity, maintaining that "...sensitivity and specificity are not proper parameters for characterizing diagnostic accuracy research... these parameters are of limited relevance to practice, and their estimation should not necessarily be pursued in diagnostic research" [33]. Note that the diagnostic "gold standard" is itself imperfect [31-33]. Our approach emphasizes the need to evaluate test characteristics in the patient population.

Neither the PLR nor *J *are calculable in the patient (target) population, and they do not convey any additional information beyond sensitivity and specificity. In a previous publication we suggested new **ratio **measures in the patient population [29]. In this manuscript we recommend the use of a new measure, PSI, as a **difference **measure that measures the overall clinical utility of a test.

The present paper suggests a similarity between *J *and the **rate difference in a cohort study**. Similarly, we suggest an analogy between PSI and the **exposure rate difference in a case control study**. These analogies have seldom been discussed in the epidemiological literature despite the obvious implications for teaching and understanding the different uses of the 2 × 2 table in etiological and clinical epidemiology.

Thus, analogously to calculations of NNT from the rate difference (RD) in follow-up studies, the reciprocal of *J*, i.e., 1J
 MathType@MTEF@5@5@+=feaafiart1ev1aqatCvAUfKttLearuWrP9MDH5MBPbIqV92AaeXatLxBI9gBaebbnrfifHhDYfgasaacH8akY=wiFfYdH8Gipec8Eeeu0xXdbba9frFj0=OqFfea0dXdd9vqai=hGuQ8kuc9pgc9s8qqaq=dirpe0xb9q8qiLsFr0=vr0=vr0dc8meaabaqaciaacaGaaeqabaqabeGadaaakeaadaWcaaqaaiabigdaXaqaaiabdQeakbaaaaa@2ECA@, can be interpreted as the number of patients needed to be examined in order to correctly detect (NND) one person with the disease [34] in a study population (Table 1) of persons with and without the known disease. NND can be helpful whenever the sensitivity and specificity, and thus *J *or PLR are used. But **NND is insensitive to variation in prevalence**, as shown in the example in Table 3: approximately two examinations are needed among persons with and without a diagnosis of a disease to detect correctly one person who has the disease, irrespective of patient characteristics and the prevalence of the diseases. Patients and physicians have little interest in a statistic that is unaffected by patient characteristics. Thus, NND should have limited clinical utility.

We suggest instead using NNP = 1PSI
 MathType@MTEF@5@5@+=feaafiart1ev1aqatCvAUfKttLearuWrP9MDH5MBPbIqV92AaeXatLxBI9gBaebbnrfifHhDYfgasaacH8akY=wiFfYdH8Gipec8Eeeu0xXdbba9frFj0=OqFfea0dXdd9vqai=hGuQ8kuc9pgc9s8qqaq=dirpe0xb9q8qiLsFr0=vr0=vr0dc8meaabaqaciaacaGaaeqabaqabeGadaaakeaadaWcaaqaaiabigdaXaqaaiabbcfaqjabbofatjabbMeajbaaaaa@311A@ to estimate the number of patients needed to be examined in the patient population (Table 2) in order to correctly predict the diagnosis of one person with a **positive **test result. This measure, the "number needed to predict," better describes the use of a diagnostic test in patient populations with a different prevalence of the disease (Table 3), and is much more meaningful for patients and physicians. Based on Sackett's example [8] and the data in Table 3, we suggest that NNP can be useful for cost assessment and policy making for specific patients at a specific risk. For example, exercise ECG could be used for patients at high risk for coronary heart disease but not for patients at low risk. Similarly, NNP estimates the number of patients who need to be tested to identify, with a **negative **test result, one patient who does not have the disease.

The interpretation of *J *is based on the assumption that the human and monetary costs associated with a false positive and a false negative in the study population (Table 1) are equal [1,25,26]. Similarly, the interpretation of PSI is based on the assumption that the human and monetary costs associated with a false positive and a false negative in the target population (Table 2) are equal. These assumptions are valid when missing the diagnosis of a disease is as important and undesirable as missing the diagnosis of the absence of a disease. If other assumptions (of unequal importance) are more appropriate, a weighted measure of PSI can be developed, similar to attempts to study weighted *J *by Faraggi [35] and by Fluss, Faraggi and Reisser [36].

*J *is one of the most important measures in receiver operating curve (ROC) analyses aimed at choosing the cutoff point at which the sensitivity and false positive rates yield the largest *J*. There is a vast literature on the use of *J *in assessing the ROC [24,25,34] in continuous diagnostic tests. Similarly, future studies should explore PSI for continuous diagnostic tests and weighted PSI. Teaching materials and software can be developed to assist physicians in using PSI, similarly to what has been accomplished by Schechter and Sheps [37] who introduced simple and accessible approaches to calculating PPV.

There are excellent computer programs available for computing *J*, notably PEPI [38]. PEPI programs also calculate the gain in certainty in the study population. Similar programs that calculate PSI could be developed with great benefit.

## Notations

P(T+) = probability of the diagnostic test being positive

P(T-) = probability of the diagnostic test being negative

P(S+) = probability of disease, i.e., prevalence

P(S-) = probability of no disease, i.e., 1-prevalence

Sensitivity = P(T+|S+)

Specificity = P(T-|S-)

PPV = positive predicted value = P(S+|T+)

NPV = negative predicted value = P(S-|T-)

*fpr *= study false positive rate = P(T+|S-) = 1-sensitivity

*fnr *= study false negative rate = P(T-|S+) = 1-specificity

PLR = positive likelihood ratio = sensitivity/(1-specificity)

NLR = negative likelihood ratio = (1-sensitivity)/specificity

PPR = positive predictive ratio = PPV/(1-NPV)

NPR = negative predictive ratio = (1-PPV)/NPV

FPR = population false positive rate = P(S-|T+) = 1-PPV

FNR = population false negative rate = P(S+|T-) = 1-NPV

*J *= Youden Index = A summary index in the study population = sensitivity+specificity-1

PSI = Ψ = Predictive Summary Index in the target population = PPV+NPV-1

RD = rate difference (in a follow-up study)

NND = number needed to detect a disease = 1/*J*

NNP = number needed to predict a diagnosis = 1/Ψ

## Appendix A

*J *is the **total **net gain in certainty when a test is applied to the study population, and is also the **average net gain **in certainty in the study population for persons with or without a disease.

Assuming that the value gain in certainty for the two study populations (persons with and without the disease) is similar, and that the false positives, *fpr*, are as undesirable as the false negatives, *fnr*, the unweighted average of the two measures is *J*:

3.23g++g−2=12[a−ca+c+d−bd+b]=J
 MathType@MTEF@5@5@+=feaafiart1ev1aqatCvAUfKttLearuWrP9MDH5MBPbIqV92AaeXatLxBI9gBaebbnrfifHhDYfgasaacH8akY=wiFfYdH8Gipec8Eeeu0xXdbba9frFj0=OqFfea0dXdd9vqai=hGuQ8kuc9pgc9s8qqaq=dirpe0xb9q8qiLsFr0=vr0=vr0dc8meaabaqaciaacaGaaeqabaqabeGadaaakeaacqaIZaWmcqGGUaGlcqaIYaGmcqaIZaWmdaWcaaqaaiabdEgaNnaaBaaaleaacqGHRaWkaeqaaOGaey4kaSIaem4zaC2aaSbaaSqaaiabgkHiTaqabaaakeaacqaIYaGmaaGaeyypa0ZaaSaaaeaacqaIXaqmaeaacqaIYaGmaaGaei4waS1aaSaaaeaacqWGHbqycqGHsislcqWGJbWyaeaacqWGHbqycqGHRaWkcqWGJbWyaaGaey4kaSYaaSaaaeaacqWGKbazcqGHsislcqWGIbGyaeaacqWGKbazcqGHRaWkcqWGIbGyaaGaeiyxa0Laeyypa0JaemOsaOeaaa@4DE3@

### Proof

Rearranging the algebraic terms:

12[a−ca+c+d−bd+b]=12[ad+ab−cd−cb+ad+dc−ab−cb(a+c)(b+d)]=122ad−2cb(a+c)(b+d)=ad−cb(a+c)(b+d)
 MathType@MTEF@5@5@+=feaafiart1ev1aaatCvAUfKttLearuWrP9MDH5MBPbIqV92AaeXatLxBI9gBaebbnrfifHhDYfgasaacH8akY=wiFfYdH8Gipec8Eeeu0xXdbba9frFj0=OqFfea0dXdd9vqai=hGuQ8kuc9pgc9s8qqaq=dirpe0xb9q8qiLsFr0=vr0=vr0dc8meaabaqaciaacaGaaeqabaqabeGadaaakqaabeqaamaalaaabaGaeGymaedabaGaeGOmaidaaiabcUfaBnaalaaabaGaemyyaeMaeyOeI0Iaem4yamgabaGaemyyaeMaey4kaSIaem4yamgaaiabgUcaRmaalaaabaGaemizaqMaeyOeI0IaemOyaigabaGaemizaqMaey4kaSIaemOyaigaaiabc2faDjabg2da9maalaaabaGaeGymaedabaGaeGOmaidaaiabcUfaBnaalaaabaGaemyyaeMaemizaqMaey4kaSIaemyyaeMaemOyaiMaeyOeI0Iaem4yamMaemizaqMaeyOeI0Iaem4yamMaemOyaiMaey4kaSIaemyyaeMaemizaqMaey4kaSIaemizaqMaem4yamMaeyOeI0IaemyyaeMaemOyaiMaeyOeI0Iaem4yamMaemOyaigabaGaeiikaGIaemyyaeMaey4kaSIaem4yamMaeiykaKIaeiikaGIaemOyaiMaey4kaSIaemizaqMaeiykaKcaaiabc2faDbqaaiabg2da9maalaaabaGaeGymaedabaGaeGOmaidaamaalaaabaGaeGOmaiJaemyyaeMaemizaqMaeyOeI0IaeGOmaiJaem4yamMaemOyaigabaGaeiikaGIaemyyaeMaey4kaSIaem4yamMaeiykaKIaeiikaGIaemOyaiMaey4kaSIaemizaqMaeiykaKcaaiabg2da9maalaaabaGaemyyaeMaemizaqMaeyOeI0Iaem4yamMaemOyaigabaGaeiikaGIaemyyaeMaey4kaSIaem4yamMaeiykaKIaeiikaGIaemOyaiMaey4kaSIaemizaqMaeiykaKcaaaaaaa@9263@

Adding and subtracting 1:

ad−bc(a+c)(b+d)+1−1=ad−bc+ab+ad+bc+cd(a+c)(b+d)−1=2ad+ab+cd(a+c)(b+d)−1=
 MathType@MTEF@5@5@+=feaafiart1ev1aaatCvAUfKttLearuWrP9MDH5MBPbIqV92AaeXatLxBI9gBaebbnrfifHhDYfgasaacH8akY=wiFfYdH8Gipec8Eeeu0xXdbba9frFj0=OqFfea0dXdd9vqai=hGuQ8kuc9pgc9s8qqaq=dirpe0xb9q8qiLsFr0=vr0=vr0dc8meaabaqaciaacaGaaeqabaqabeGadaaakeaadaWcaaqaaiabdggaHjabdsgaKjabgkHiTiabdkgaIjabdogaJbqaaiabcIcaOiabdggaHjabgUcaRiabdogaJjabcMcaPiabcIcaOiabdkgaIjabgUcaRiabdsgaKjabcMcaPaaacqGHRaWkcqaIXaqmcqGHsislcqaIXaqmcqGH9aqpdaWcaaqaaiabdggaHjabdsgaKjabgkHiTiabdkgaIjabdogaJjabgUcaRiabdggaHjabdkgaIjabgUcaRiabdggaHjabdsgaKjabgUcaRiabdkgaIjabdogaJjabgUcaRiabdogaJjabdsgaKbqaaiabcIcaOiabdggaHjabgUcaRiabdogaJjabcMcaPiabcIcaOiabdkgaIjabgUcaRiabdsgaKjabcMcaPaaacqGHsislcqaIXaqmcqGH9aqpdaWcaaqaaiabikdaYiabdggaHjabdsgaKjabgUcaRiabdggaHjabdkgaIjabgUcaRiabdogaJjabdsgaKbqaaiabcIcaOiabdggaHjabgUcaRiabdogaJjabcMcaPiabcIcaOiabdkgaIjabgUcaRiabdsgaKjabcMcaPaaacqGHsislcqaIXaqmcqGH9aqpaaa@7B43@

2ad+ab+cd(a+c)(b+d)−1=a(b+d)+d(a+c)(a+c)(b+d)−1==aa+c+db+d−1=sensitivity+specificity−1=J
 MathType@MTEF@5@5@+=feaafiart1ev1aaatCvAUfKttLearuWrP9MDH5MBPbIqV92AaeXatLxBI9gBaebbnrfifHhDYfgasaacH8akY=wiFfYdH8Gipec8Eeeu0xXdbba9frFj0=OqFfea0dXdd9vqai=hGuQ8kuc9pgc9s8qqaq=dirpe0xb9q8qiLsFr0=vr0=vr0dc8meaabaqaciaacaGaaeqabaqabeGadaaakqaabeqaamaalaaabaGaeGOmaiJaemyyaeMaemizaqMaey4kaSIaemyyaeMaemOyaiMaey4kaSIaem4yamMaemizaqgabaGaeiikaGIaemyyaeMaey4kaSIaem4yamMaeiykaKIaeiikaGIaemOyaiMaey4kaSIaemizaqMaeiykaKcaaiabgkHiTiabigdaXiabg2da9maalaaabaGaemyyaeMaeiikaGIaemOyaiMaey4kaSIaemizaqMaeiykaKIaey4kaSIaemizaqMaeiikaGIaemyyaeMaey4kaSIaem4yamMaeiykaKcabaGaeiikaGIaemyyaeMaey4kaSIaem4yamMaeiykaKIaeiikaGIaemOyaiMaey4kaSIaemizaqMaeiykaKcaaiabgkHiTiabigdaXiabg2da9aqaaiabg2da9maalaaabaGaemyyaegabaGaemyyaeMaey4kaSIaem4yamgaaiabgUcaRmaalaaabaGaemizaqgabaGaemOyaiMaey4kaSIaemizaqgaaiabgkHiTiabigdaXiabg2da9iabdohaZjabdwgaLjabd6gaUjabdohaZjabdMgaPjabdsha0jabdMgaPjabdAha2jabdMgaPjabdsha0jabdMha5jabgUcaRiabdohaZjabdchaWjabdwgaLjabdogaJjabdMgaPjabdAgaMjabdMgaPjabdogaJjabdMgaPjabdsha0jabdMha5jabgkHiTiabigdaXiabg2da9iabdQeakbaaaa@91BA@

## Appendix B

PSI is the **total **net gain in certainty when a test is applied to the target population and is also the average net gain in certainty for persons with a positive or negative test:

12[a−ca+c+d−bd+b]=12[ad+ab−cd−cb+ad+dc−ab−cb(a+c)(b+d)]=122ad−2cb(a+c)(b+d)=ad−cb(a+c)(b+d)
 MathType@MTEF@5@5@+=feaafiart1ev1aaatCvAUfKttLearuWrP9MDH5MBPbIqV92AaeXatLxBI9gBaebbnrfifHhDYfgasaacH8akY=wiFfYdH8Gipec8Eeeu0xXdbba9frFj0=OqFfea0dXdd9vqai=hGuQ8kuc9pgc9s8qqaq=dirpe0xb9q8qiLsFr0=vr0=vr0dc8meaabaqaciaacaGaaeqabaqabeGadaaakqaabeqaamaalaaabaGaeGymaedabaGaeGOmaidaaiabcUfaBnaalaaabaGaemyyaeMaeyOeI0Iaem4yamgabaGaemyyaeMaey4kaSIaem4yamgaaiabgUcaRmaalaaabaGaemizaqMaeyOeI0IaemOyaigabaGaemizaqMaey4kaSIaemOyaigaaiabc2faDjabg2da9maalaaabaGaeGymaedabaGaeGOmaidaaiabcUfaBnaalaaabaGaemyyaeMaemizaqMaey4kaSIaemyyaeMaemOyaiMaeyOeI0Iaem4yamMaemizaqMaeyOeI0Iaem4yamMaemOyaiMaey4kaSIaemyyaeMaemizaqMaey4kaSIaemizaqMaem4yamMaeyOeI0IaemyyaeMaemOyaiMaeyOeI0Iaem4yamMaemOyaigabaGaeiikaGIaemyyaeMaey4kaSIaem4yamMaeiykaKIaeiikaGIaemOyaiMaey4kaSIaemizaqMaeiykaKcaaiabc2faDbqaaiabg2da9maalaaabaGaeGymaedabaGaeGOmaidaamaalaaabaGaeGOmaiJaemyyaeMaemizaqMaeyOeI0IaeGOmaiJaem4yamMaemOyaigabaGaeiikaGIaemyyaeMaey4kaSIaem4yamMaeiykaKIaeiikaGIaemOyaiMaey4kaSIaemizaqMaeiykaKcaaiabg2da9maalaaabaGaemyyaeMaemizaqMaeyOeI0Iaem4yamMaemOyaigabaGaeiikaGIaemyyaeMaey4kaSIaem4yamMaeiykaKIaeiikaGIaemOyaiMaey4kaSIaemizaqMaeiykaKcaaaaaaa@9263@

Assuming that the value gain in certainty for the two target populations (persons with a positive test and without a positive test) is similar, and that the false positives (FPR) are as undesirable as the false negatives (FNR), the unweighted average of the two measures is PSI.

### Proof

Rearranging the algebraic terms:

12[A−BA+B+D−CD+C]=12[AD+AC−BD−BC+AD−AC+BD−BC(A+B)(C+D)]=122AD−2BC(A+B)(C+D)=AD−BC(A+B)(C+D)=
 MathType@MTEF@5@5@+=feaafiart1ev1aaatCvAUfKttLearuWrP9MDH5MBPbIqV92AaeXatLxBI9gBaebbnrfifHhDYfgasaacH8akY=wiFfYdH8Gipec8Eeeu0xXdbba9frFj0=OqFfea0dXdd9vqai=hGuQ8kuc9pgc9s8qqaq=dirpe0xb9q8qiLsFr0=vr0=vr0dc8meaabaqaciaacaGaaeqabaqabeGadaaakqaabeqaamaalaaabaGaeGymaedabaGaeGOmaidaaiabcUfaBnaalaaabaGaemyqaeKaeyOeI0IaemOqaieabaGaemyqaeKaey4kaSIaemOqaieaaiabgUcaRmaalaaabaGaemiraqKaeyOeI0Iaem4qameabaGaemiraqKaey4kaSIaem4qameaaiabc2faDjabg2da9maalaaabaGaeGymaedabaGaeGOmaidaaiabcUfaBnaalaaabaGaemyqaeKaemiraqKaey4kaSIaemyqaeKaem4qamKaeyOeI0IaemOqaiKaemiraqKaeyOeI0IaemOqaiKaem4qamKaey4kaSIaemyqaeKaemiraqKaeyOeI0IaemyqaeKaem4qamKaey4kaSIaemOqaiKaemiraqKaeyOeI0IaemOqaiKaem4qameabaGaeiikaGIaemyqaeKaey4kaSIaemOqaiKaeiykaKIaeiikaGIaem4qamKaey4kaSIaemiraqKaeiykaKcaaiabc2faDbqaaiabg2da9maalaaabaGaeGymaedabaGaeGOmaidaamaalaaabaGaeGOmaiJaemyqaeKaemiraqKaeyOeI0IaeGOmaiJaemOqaiKaem4qameabaGaeiikaGIaemyqaeKaey4kaSIaemOqaiKaeiykaKIaeiikaGIaem4qamKaey4kaSIaemiraqKaeiykaKcaaiabg2da9maalaaabaGaemyqaeKaemiraqKaeyOeI0IaemOqaiKaem4qameabaGaeiikaGIaemyqaeKaey4kaSIaemOqaiKaeiykaKIaeiikaGIaem4qamKaey4kaSIaemiraqKaeiykaKcaaiabg2da9aaaaa@8869@

Adding and subtracting 1:

AD−BC(A+B)(C+D)+1−1=AD−BC+AC+AD+BC+BD(A+B)(C+D)==2AD+AC+BD(A+B)(C+D)−1=
 MathType@MTEF@5@5@+=feaafiart1ev1aaatCvAUfKttLearuWrP9MDH5MBPbIqV92AaeXatLxBI9gBaebbnrfifHhDYfgasaacH8akY=wiFfYdH8Gipec8Eeeu0xXdbba9frFj0=OqFfea0dXdd9vqai=hGuQ8kuc9pgc9s8qqaq=dirpe0xb9q8qiLsFr0=vr0=vr0dc8meaabaqaciaacaGaaeqabaqabeGadaaakqaabeqaamaalaaabaGaemyqaeKaemiraqKaeyOeI0IaemOqaiKaem4qameabaGaeiikaGIaemyqaeKaey4kaSIaemOqaiKaeiykaKIaeiikaGIaem4qamKaey4kaSIaemiraqKaeiykaKcaaiabgUcaRiabigdaXiabgkHiTiabigdaXiabg2da9maalaaabaGaemyqaeKaemiraqKaeyOeI0IaemOqaiKaem4qamKaey4kaSIaemyqaeKaem4qamKaey4kaSIaemyqaeKaemiraqKaey4kaSIaemOqaiKaem4qamKaey4kaSIaemOqaiKaemiraqeabaGaeiikaGIaemyqaeKaey4kaSIaemOqaiKaeiykaKIaeiikaGIaem4qamKaey4kaSIaemiraqKaeiykaKcaaiabg2da9aqaaiabg2da9maalaaabaGaeGOmaiJaemyqaeKaemiraqKaey4kaSIaemyqaeKaem4qamKaey4kaSIaemOqaiKaemiraqeabaGaeiikaGIaemyqaeKaey4kaSIaemOqaiKaeiykaKIaeiikaGIaem4qamKaey4kaSIaemiraqKaeiykaKcaaiabgkHiTiabigdaXiabg2da9aaaaa@71F3@

=A(C+D)+D(A+B)(A+B)(C+D)−1=
 MathType@MTEF@5@5@+=feaafiart1ev1aaatCvAUfKttLearuWrP9MDH5MBPbIqV92AaeXatLxBI9gBaebbnrfifHhDYfgasaacH8akY=wiFfYdH8Gipec8Eeeu0xXdbba9frFj0=OqFfea0dXdd9vqai=hGuQ8kuc9pgc9s8qqaq=dirpe0xb9q8qiLsFr0=vr0=vr0dc8meaabaqaciaacaGaaeqabaqabeGadaaakeaacqGH9aqpdaWcaaqaaiabdgeabjabcIcaOiabdoeadjabgUcaRiabdseaejabcMcaPiabgUcaRiabdseaejabcIcaOiabdgeabjabgUcaRiabdkeacjabcMcaPaqaaiabcIcaOiabdgeabjabgUcaRiabdkeacjabcMcaPiabcIcaOiabdoeadjabgUcaRiabdseaejabcMcaPaaacqGHsislcqaIXaqmcqGH9aqpaaa@4663@

=AA+B+DC+D−1=PPV+NPV−1=PSI
 MathType@MTEF@5@5@+=feaafiart1ev1aaatCvAUfKttLearuWrP9MDH5MBPbIqV92AaeXatLxBI9gBaebbnrfifHhDYfgasaacH8akY=wiFfYdH8Gipec8Eeeu0xXdbba9frFj0=OqFfea0dXdd9vqai=hGuQ8kuc9pgc9s8qqaq=dirpe0xb9q8qiLsFr0=vr0=vr0dc8meaabaqaciaacaGaaeqabaqabeGadaaakeaacqGH9aqpdaWcaaqaaiabdgeabbqaaiabdgeabjabgUcaRiabdkeacbaacqGHRaWkdaWcaaqaaiabdseaebqaaiabdoeadjabgUcaRiabdseaebaacqGHsislcqaIXaqmcqGH9aqpcqqGqbaucqqGqbaucqqGwbGvcqGHRaWkcqqGobGtcqqGqbaucqqGwbGvcqGHsislcqaIXaqmcqGH9aqpcqqGqbaucqqGtbWucqqGjbqsaaa@47DF@

## References

[B1] Youden EJ (1950). Index for rating diagnostic tests. Cancer.

[B2] Connell FA, Koepsell TD (1985). Measures of gain in certainty from diagnostic test. American Journal of Epidemiology.

[B3] Salmi LR (1986). Re: Measures of gain in certainty from a diagnostic test. American Journal of Epidemiology.

[B4] Weinstein MC, Finberg HV (1980). Clinical Decision Analysis.

[B5] Altman DG (1991). Practical Statistics for Medical Research.

[B6] Riffenburgh RH (1993). Statistics in Medicine.

[B7] Hirsch RP, Riegelman RK (1996). Statistical Operations.

[B8] Sackett DL, Haynes RB, Guyatt GH, Tugwell P (1991). Clinical Epidemiology.

[B9] Kraemer HC (1992). Evaluation of Medical Tests. Objective and Quantitative Guidelines.

[B10] Beaglehole R, Bonita R, Kjellstrom T (1993). Basic Epidemiology.

[B11] Bradley GW (1993). Disease Diagnosis and Decision.

[B12] Essex-Sorlie D (1995). Medical Biostatistics and Epidemiology.

[B13] Jenicek M (1995). Epidemiology The Logic of Modern Medicine.

[B14] Wassertheil S (1995). Biostatistics and Epidemiology.

[B15] Weiss NS (1996). Clinical Epidemiology.

[B16] Rothman KJ, Greenland S (1998). Modern Epidemiology.

[B17] Silva SI (1999). Cancer Epidemiology: Principles and Methods.

[B18] Feinstein AR (2002). Principles of Medical Statistics.

[B19] Riegelman RK (2000). Studying a Study and Testing a Test.

[B20] Dawson B, Trapp RG (2001). Basic and Clinical Biostatistics.

[B21] Greenberg RS, Daniels SR, Flanders WD, Eley JW, Boring JR (2001). Medical Epidemiology.

[B22] Knottnerus JA, van Weel C, Knottnerus JA (2002). General introduction: Evaluation of diagnostic procedures. The Evidence Base of Clinical Diagnosis.

[B23] Sackett D, Haynes RB, Knottnerus JA (2002). The architecture of diagnostic research. The Evidence Base of Clinical Diagnosis.

[B24] Habbema JD, Eijkemans R, Krijnen P, Knottnerus JA, Knottnerus JA (2002). Analysis of data on the accuracy of diagnostic tests. The Evidence Base of Clinical Diagnosis.

[B25] Zhou XH, Obuchoedki NA, McClish DK (2002). Statistical Methods in Diagnostic Medicine.

[B26] Pepe MS (2003). The Statistical Evaluation of Medical Tests for Classification and Prediction. Oxford Statistical Science Series 28.

[B27] Fleiss JL (1981). Statistical Methods for Rates and Proportions.

[B28] Reid MC, Lane DA, Feinstein AR (1998). Academic calculation versus clinical judgments: Practicing physicians' use of quantitative measures of test accuracy. American Journal of Medicine.

[B29] Linn S (2005). New patient-oriented diagnostic test characteristics analogous to the likelihood ratios convey information on trustfulness. Clinical Epidemiology.

[B30] Zweig MH, Campbell G (1993). Receiver-operating characteristics (ROC) plots: A fundamental evaluation tool in clinical medicine. Clinical Chemistry.

[B31] Ransohoff DF (2002). Challenges and opportunities in evaluating diagnostic tests. Journal of Clinical Epidemiology.

[B32] Marshall KG (1996). Prevention. How much harm? How much benefit? 3. Physical, psychological and social harm. Canadian Medical Association Journal.

[B33] Moons KGM, Harrell FE (2003). Sensitivity and specificity should be de-emphasized in diagnostic accuracy studies. Academic Radiology.

[B34] Bandolier (1996). How good is the test. http://www.jr2.ox.ac.uk/bandolier/band27/b27-2.html.

[B35] Faraggi D (2003). Adjusting receiver operating characteristic curve and related indices for covariates. Journal of the Royal Statistical Society: Series D (the Statistician).

[B36] Fluss, Ronen Estimation of the Youden index and the associated Cut-Off Point. MSc Thesis supervised by David Faraggi, Benjamin Reisser.

[B37] Schechter MT, Sheps SB (1985). Diagnostic testing revisited: pathways through uncertainty. Canadian Medical Association Journal.

[B38] Abramson JH, Paul Gahlinger Computer Programs for Epidemiologists – PEPI Version 4.0.

